# Tumor-associated macrophages, potential targets for cancer treatment

**DOI:** 10.1186/s40364-017-0106-7

**Published:** 2017-08-08

**Authors:** Li Yang, Yi Zhang

**Affiliations:** 1 Biotherapy Center and Cancer Center, The First Affiliated Hospital of Zhengzhou University, No.1 Jianshe East Road, Zhengzhou, Henan Province 450052 China; 2 School of Life Sciences, Zhengzhou University, No.100 Kexue Road, Zhengzhou, Henan Province 450001 China

**Keywords:** Tumor-associated macrophages (TAMs), Tumor microenvironment, Therapeutic target, Cancer treatment, Protumoral activities

## Abstract

The fact that various immune cells, including macrophages, can be found in tumor tissues has long been known. With the introduction of concept that macrophages differentiate into a classically or alternatively activated phenotype, the role of tumor-associated macrophages (TAMs) is now beginning to be elucidated. TAMs act as “protumoral macrophages”, contributing to disease progression. As the relationship between TAMs and malignant tumors becomes clearer, TAMs are beginning to be seen as potential therapeutic targets in these cases. In this review, we will discuss how TAMs can be used as therapeutic targets of cancer in clinics.

## Background

Non-resolving inflammation in a tumor microenvironment is a hallmark of cancer. Leukocytes, fibroblasts, and vascular endothelial cells together form a tumor microenvironment, with immune cells representing its major component. These immune cells interact with tumor cells to influence the initiation, growth, and metastasis of tumors. Tumor-associated macrophages (TAMs), specifically, are often prominent immune cells that orchestrate various factors in the tumor microenvironment [1, 2].

In general, TAMs are thought to more closely resemble M2-polarized macrophages [3], also known as alternatively activated macrophages, which are activated by helper T cell 2 cytokines (e.g., interleukin (IL) -4, IL-10, and IL-13). TAMs play an important role in connecting inflammation with cancer. TAMs can promote proliferation, invasion, and metastasis of tumor cells, stimulate tumor angiogenesis, and inhibit antitumor immune response mediated by T cells, followed by the promotion of tumor progression [3]. There are strong evidences of tumor promotion by TAMs in different cancer models, and an increased TAM prevalence correlates with low survival rates in many human cancers.

With the unraveling of the relationship between TAMs and malignant tumors, TAMs are now being recognized as potential therapeutic targets for cancer. Targeting TAMs is a novel strategy for treatment of cancers. In this review, we summarize how TAMs are used as therapeutic targets in cancers.

### Limiting monocyte recruitment

One strategy for targeting TAMs is to block monocyte recruitment into tumor tissues. Targeting the chemokine (C-C motif) ligand 2 (CCL2) - chemokine (C-C motif) receptor (CCR2) axis is promising due to its important role in monocyte recruitment in tumors. A CCL2-blocking agent (carlumab, CNTO88) has been shown to inhibit the growth of several cancers in animal models. A phase II study of carlumab in metastatic castration-resistant prostate cancer patients showed that this antibody was well-tolerated, but that neither blocked the CCL2/CCR2 axis nor showed antitumor activity as a single agent in these metastatic cancer patients [4] (NCT00992186, Table 1). Similar results from Brana et al. showed that carlumab in combination with four chemotherapy regimens for the treatment of patients with solid tumors was well-tolerated, although no long-term suppression of serum CCL2 or significant tumor responses were observed [5] (NCT01204996, Table 1). However, according to the results of other study, carlumab was well-tolerated with evidence of transient CCL2 suppression and preliminary antitumor activity [6] (NCT00537368, Table 1).

**Table 1:** Clinical trials of agents that target TAMs for cancer treatment

Action	Agent name	Target	Status	Phase	Tumor type	Effect	Trial number
Limiting monocyte recruitment	Carlumab	CCL2	Completed	II	Metastatic castration-resistant prostate cancer	Well-tolerated, no antitumor activity as a single agent	NCT00992186
			Completed	Ib	Solid tumors	Well-tolerated, no long-term suppression of serum CCL2 or significant tumor responses	NCT01204996
			Completed	I	Solid tumors	Transient CCL2 suppression, preliminary antitumor activity	NCT00537368
	PF-04136309	CCR2	Completed	Ib	Locally advanced pancreatic cancer	Safe and tolerable, objective tumor response	NCT01413022
	MLN1202	CCR2	Completed	II	Bone metastases	uNTX response rate: 14/43	NCT01015560
Targeting TAM activation	MCS110	CSF1	Recruiting	II	Advanced triple negative breast cancer	NA	NCT02435680
			Recruiting	Ib/II	Advanced malignancies	NA	NCT01643850
			Terminated	I/II	Prostate cancer, bone metastases	NA	NCT00757757
	IMC-CS4	CSF1R	Recruiting	I	Advanced solid tumors	NA	NCT01346358
			Recruiting	I	Advanced, refractory breast or prostate cancer	NA	NCT02265536
	AMG 820	CSF1R	Completed	I	Advanced solid tumors	NA	NCT01444404
			Recruiting	I/II	Pancreatic cancer, colorectal cancer, non-small cell lung cancer	NA	NCT02713529
	PLX7486	CSF1R	Recruiting	I	Advanced solid tumors	NA	NCT01804530
	PLX3397	CSF1R	Completed	II	Recurrent glioblastoma	Well tolerated, no efficacy	NCT01349036
			Completed	II	Relapsed or refractory Hodgkin’s lymphoma	Safe, response rate: 1/20	NCT01217229
			Completed	II	Advanced castration-resistant prostate cancer	NA	NCT01499043
			Recruiting	I/II	Sarcoma, malignant peripheral nerve sheath tumors	NA	NCT02584647
			Recruiting	II	Advanced melanoma, other solid tumors	NA	NCT02452424
			Recruiting	Ib/II	Metastatic breast cancer	NA	NCT01596751
			Recruiting	I/II	Refractory leukemias, solid tumors	NA	NCT02390752
			Recruiting	I	Advanced solid tumors	NA	NCT01525602
	Alemtuzumab	CD52	Terminated	I	Ovarian, fallopian, or primary peritoneal cancers	NA	NCT00637390
			Completed	II	Kidney cancer	NA	NCT00073879
Reprogramming TAMs to antitumor macrophages	ChiLob 7/4	CD40	Completed	I	Advanced malignancies refractory to conventional anti-cancer treatment	Safe, activate B and NK cells	NCT01561911
	(GM.CD40L) vaccine with CCL21	CD40	Active, not recruiting	I/II	Lung cancer	NA	NCT01433172
	Tremelimumab and CP-870, 893	CD40	Active, not recruiting	I	Metastatic melanoma	NA	NCT01103635
	WP1066	STAT3	Not yet recruiting	I	Recurrent malignant glioma and brain metastases	NA	NCT01904123
	AZD9150 (ISIS-STAT3Rx)	STAT3	Completed	I/Ib	Advanced/metastatic hepatocellular carcinoma	NA	NCT01839604
	β-glucan	MAPK	Completed	II	Stage IV KRAS-mutant colorectal cancer	Compelling, albeit modest, clinical activity	NCT00912327
			Recruiting	I	Neuroblastoma	NA	NCT00911560
			Active, not recruiting	I	Metastatic neuroblastoma	NA	NCT00492167
	Hu5F9-G4	CD47	Recruiting	I	Solid tumor	NA	NCT02216409
	CC-90002 and Rituximab	CD47	Recruiting	I	Hematologic neoplasms	NA	NCT02367196

Sanford et al. demonstrates that a CCR2 antagonist (PF-04136309) can block the mobilization of CCR2^+^ monocytes from bone marrow to tumors in a mouse model of pancreatic cancer and can lead to TAM depletion, causing the inhibition of tumor growth and distant metastasis [7]. PF-04136309, in combination with FOLFIRINOX chemotherapy, was used in a phase Ib trial (NCT01413022, Table 1). This therapy was found safe and tolerable with an objective tumor response [8]. Moreover, the efficiency of the humanized antibody specific for CCR2 (MLN1202) was determined in a clinical investigation (NCT01015560, Table 1).

Treatment with systemic CD11b-neutralizing monoclonal antibodies has been shown to prevent the recruitment of myeloid cells to tumors. It has been shown that the use of Mac-1 (CD11b/CD18) antibodies leads to an improved response to radiation therapy in squamous cell carcinoma xenografts of mice, which is accompanied by reduced infiltration of myeloid cells expressing matrix metallopeptidase-9 and S100A8 inside tumors [9].

Because targeting monocytes, prior to being recruited to tumors, has been effective in various cancer models and partial clinical trials, TAMs can be directly targeted as well by other approaches once they invade tumors.

### Targeting the activation of TAMs

TAMs can be targeted at the level of activation using various strategies. Colony-stimulating factor 1 (CSF1)/CSF1 receptor (CSF1R) signaling is critical for the generation of monocyte progenitors in bone marrow and TAM polarization in tumor tissues. For these reasons, CSF1/CSF1R signaling is an attractive target for cancer treatment. Genetic loss of CSF1 results in significantly reduced metastasis and delayed tumor progression in breast and neuroendocrine tumor models [10]. Based on these results, several clinical trials of CSF1/CSF1R inhibitors have been completed or are ongoing (Table 1).

Macrophage surface markers can act as useful therapeutic targets. Mannose receptor CD206 can be exploited as a macrophage-specific target. A single-chain peptide bound to the CD206 receptor was attached to nanobodies that can selectively target CD206^+^ TAMs [11]. Legumain, a stress protein and a member of the asparagine endopeptidase family, can serve as an efficient therapeutic target when overexpressed in TAMs [12]. Targeting surface markers such as scavenger receptor A and CD52 by using immunotoxin-conjugated monoclonal antibodies (mAbs) has been investigated in ovarian cancer [13]. Moreover, the efficiency of alemtuzumab (anti-CD52 antibody) as a tumor treatment in ongoing clinical trials is under investigation (NCT00637390, NCT00073879, Table 1).

Trabectedin (ET743, Yondelis®) was shown to decrease the number of TAMs in tumor tissues by inducing apoptosis of monocytes and macrophages [14]. Based on the favorable results of several phase I, II, and III clinical trials, trabectedin has gained full marketing approval from the European Commission for use in the treatment of ovarian cancer and soft tissue sarcomas and FDA approval in 2015 for use in unresectable or metastatic liposarcoma or leiomyosarcoma [15].

### Reprogramming TAMs to antitumor macrophages

As discussed above, one of the key features of macrophages is their plasticity, which enables them to change their phenotype in the tumor microenvironment. Thus, reprogramming TAMs to an antitumor phenotype is an attractive therapeutic strategy. The results of our previous study showed that pseudomonas aeruginosa mannose-sensitive hemagglutinin, which is used in MPE treatment, re-educated CD163^+^ TAMs to M1 macrophages in MPE, suggesting that reprogramming CD163^+^ TAMs can be served as a potential therapeutic strategy of MPE [16].

Nanoparticles are gradually used in polarization of TAMs into antitumor macrophages. Recently, Zanganeh et al. found that ferumoxytol significantly inhibited growth of subcutaneous adenocarcinomas in mice, and this tumor growth inhibition was accompanied by an increase in pro-inflammatory M1 macrophages in tumor tissues [17]. Recent data suggest that bioconjugated manganese dioxide nanoparticles enhance the responses of chemotherapy by inducing TAM toward M1-like phenotype [18]. Synthesized nanoparticles with IL-12 payload can reverse macrophages to antitumor function [19].

CD40 is a surface marker of macrophages that can be used to inhibit cytotoxic functions. The combination of a CD40 agonist with gemcitabine in unresectable pancreatic cancer resulted in regression of tumors by promoting antitumor macrophages [20]. ChiLob 7/4 is an intermediate CD40 agonist and chimeric IgG1, which was also shown to induce pro-inflammatory cytokines, with promising results in CD40-expressing solid tumors and diffuse large B-cell lymphoma resistant to conventional therapy in a phase I clinical trial [21] (NCT01561911, Table 1). Other clinical trials of molecules targeting CD40 for cancer treatment are ongoing (NCT01433172, NCT01103635, Table 1).

Activation of the nuclear factor κB pathway also plays an important role in polarization of TAMs to an antitumor phenotype using Toll-like receptor agonists, anti-CD40 mAbs and IL-10 mAbs [22]. In addition, regulation of STAT1 activity is an attractive strategy to induce an antitumor phenotype in macrophages because of the increase production of IL-12 in a murine carcinoma model. A small molecule inhibitor of STAT3 (WP1066) was found to reverse immune tolerance in patients with malignant gliomas and to selectively induce the expression of costimulatory molecules CD80, CD86, and IL-12 on peripheral and tumor-infiltrating macrophages [23]. An investigation of this agent to treat recurrent malignant gliomas and brain metastasis are ongoing (NCT01904123, Table 1).

Thymosin-α is an immunomodulating hormone that can reeducate TAMs into dendritic cells, which participate in antitumor host responses and produce high level of pro-inflammatory cytokines. Nanodelivery of thymosin-α is a feasible approach to increase immune activity in cancer patients. Moreover, several clinical trials have confirmed that thymosin-α prolongs survival in patients with metastatic melanomas and advanced non-small cell lung cancers [24].

β-glucan, a yeast-derived polysaccharide, has been shown to differentiate TAMs into an M1 phenotype, and is a potent immunomodulator with anti-cancer properties. The use of β-glucan is currently under investigation in a phase I clinical trial of patients with neuroblastoma [25] (NCT00911560, Table 1). In another clinical trial, a β-glucan polymer (PGG) showed compelling but modest activity in a phase II multi-cancer study [26] (NCT00912327, Table 1). Furthermore, the efficiency of β-glucan is currently under phase I clinical investigation (NCT00492167, Table 1).

In addition, CD47 has been identified as an important “don’t eat me” signal expressed on malignant cells [27]. Blockade of the CD47:SIRP-α axis between tumor cells and macrophages increases tumor cell phagocytosis in both solid tumors and hematological malignancies. Two phase I dose escalation trials are currently underway with anti-CD47 antibodies as a monotherapy for the treatment of advanced solid tumors and hematological cancers [27] (NCT02216409, NCT02367196, Table 1). CD47 blocking agents are expected to be well tolerated, efficacious and broadly applicable for cancer therapies.

### TAMs as a carry of anti-cancer drugs

TAMs can also be used as a carry of anti-cancer drugs, which is one of the most promising strategies of cancer treatment. It has been reported that macrophages can actively internalize gold nanoshells and deliver them into hypoxic regions of tumors [28], inducing cancer cell death around these macrophages. Wang et al. found that macrophages loaded by a magnetic shell combined with topoisomerase I inhibitor SN38 could deliver into the tumor site and exert an anti-cancer effect [29]. In addition, similar combination therapy was showed by Ikehara et al. [30]. Nanoparticles coated with mannose and loaded with 5-fluorouracil were internalized by macrophages. The tumor growth inhibition was observed when an electromagnetic field was applied in a mouse intraperitoneal metastatic model.

### Targeting TAMs in combination with standard therapies

Radiotherapy and chemotherapy are useful treatments in many cancers, and studies have shown that infiltrated-myeloid increases after irradiation. However, the interaction between tumor cells and stroma after these therapies remains poorly defined. DNA damage, cell death, and increased hypoxia have been observed in tumors after radiotherapy, which has been shown to lead to macrophage recruitment and promote tumor progression in animal models [31]. Therefore, it is essential to combine TAM targeting with standard therapies for effective tumor treatment.

The hypoxia-inducible factor-1 (HIF-1) pathway is stimulated by radiation-induced tumor hypoxia and the HIF-1 inhibitor can result in decreased infiltration of myeloid cells into tumors [32]. Even more strikingly, blocking CSF1R signaling appears to enhance the efficacy of several other standard therapies. As such, CSF1R blockade has been shown to increase the efficacy of chemotherapy for pancreatic tumors [33].

## Conclusions

Targeting TAMs is a promising strategy for cancer treatment. Recent ongoing experimental, pre-clinical, and clinical studies of TAMs have shown encouraging progress. We believe that TAM-targeted therapies would be applied in cancer patients in the future.

## Abbreviations

CCL2: Chemokine (C-C motif) ligand 2
CCR2: Chemokine (C-C motif) receptor 2
CSF1: Colony-stimulating factor
CSF1R: CSF1 receptor
HIF-1: Hypoxia-inducible factor 1
IL: Interleukin
TAMs: Tumor-associated macrophages
